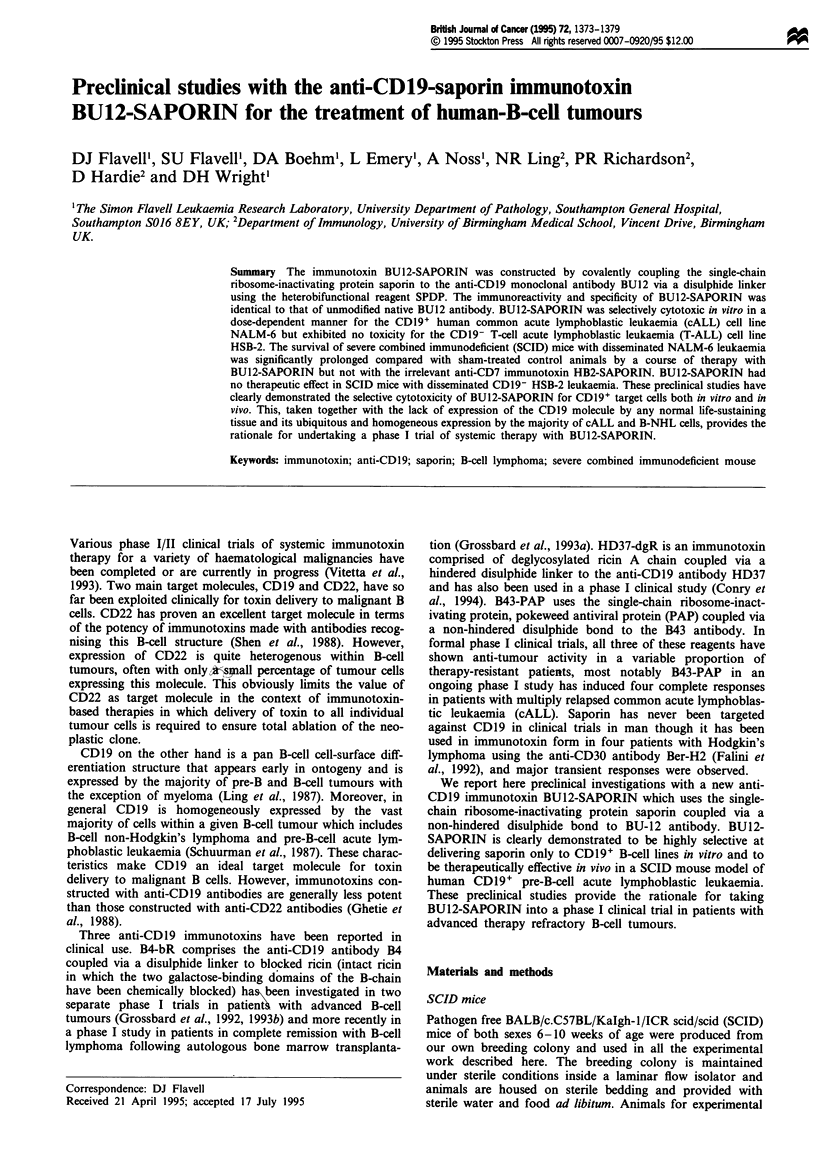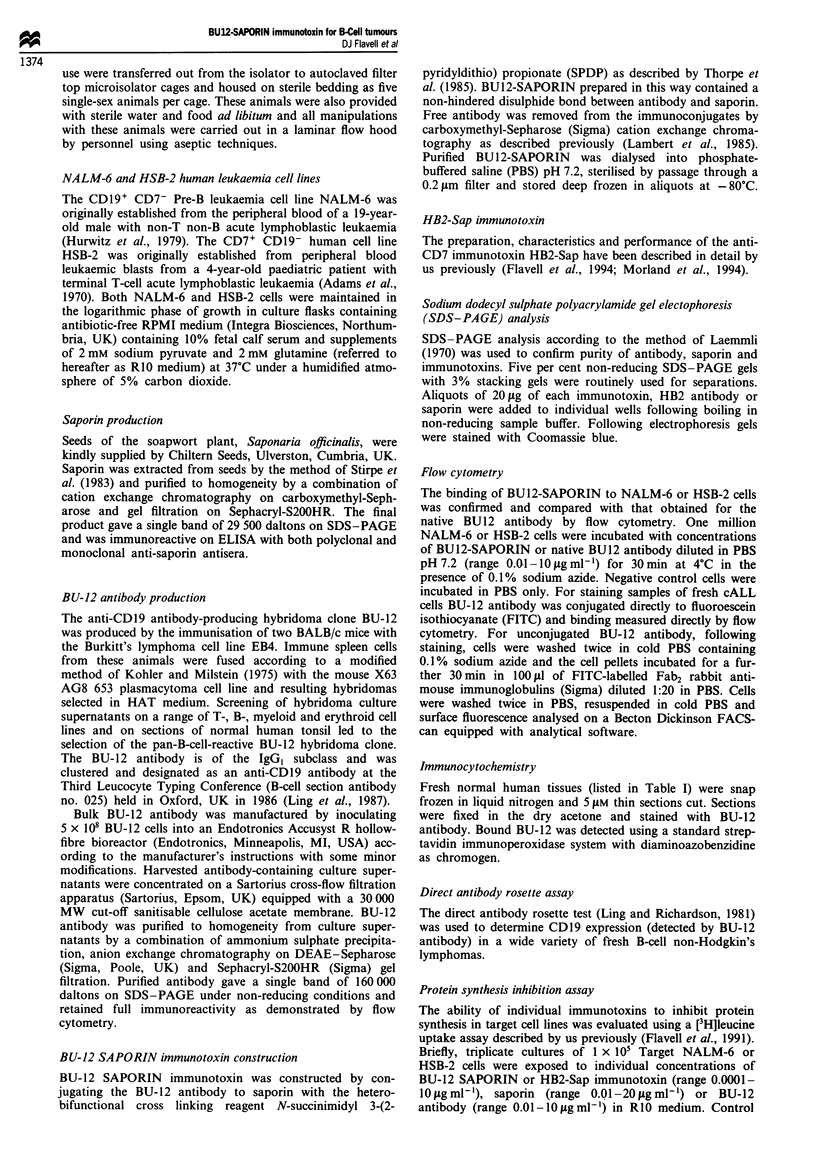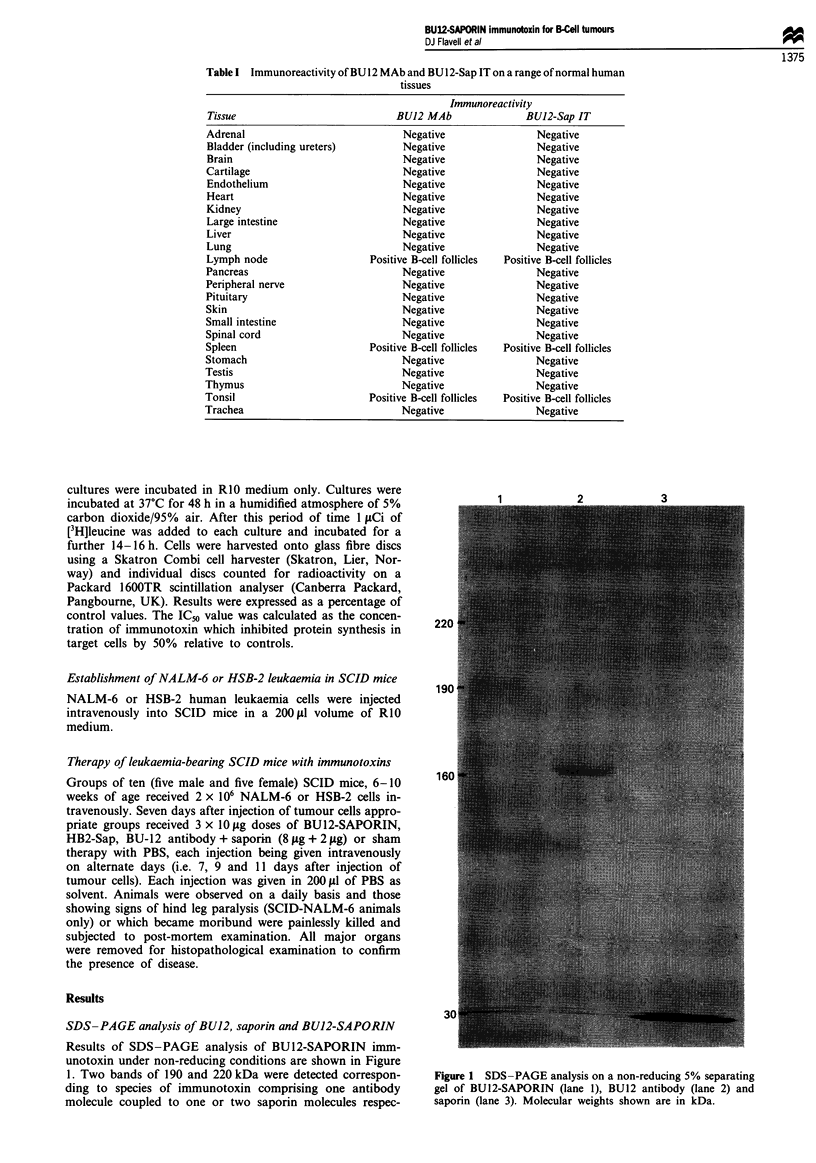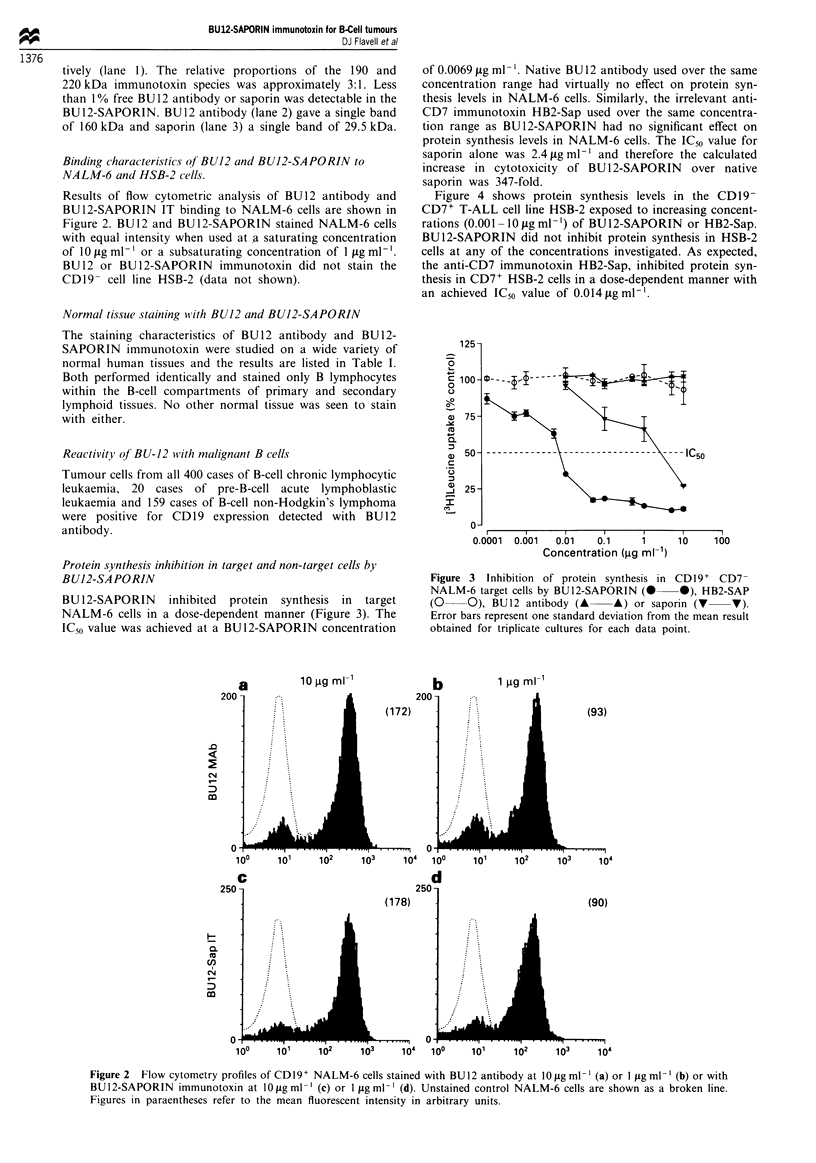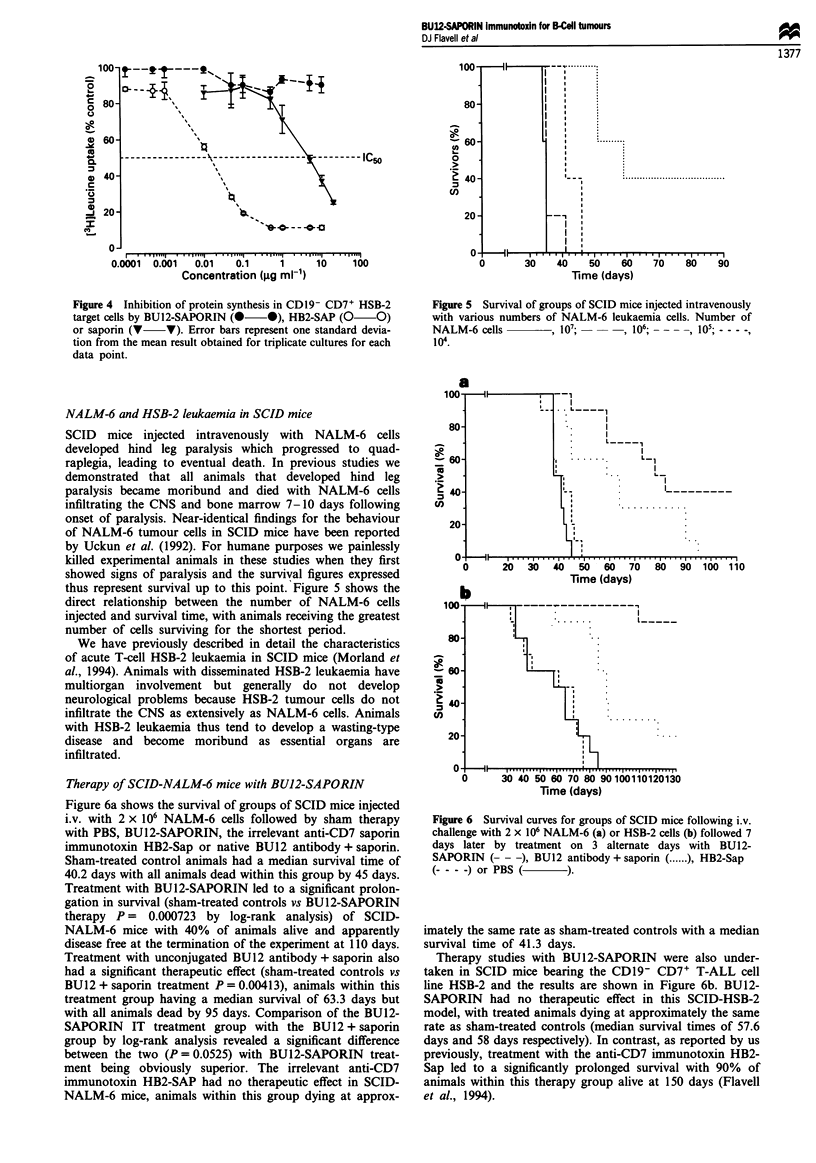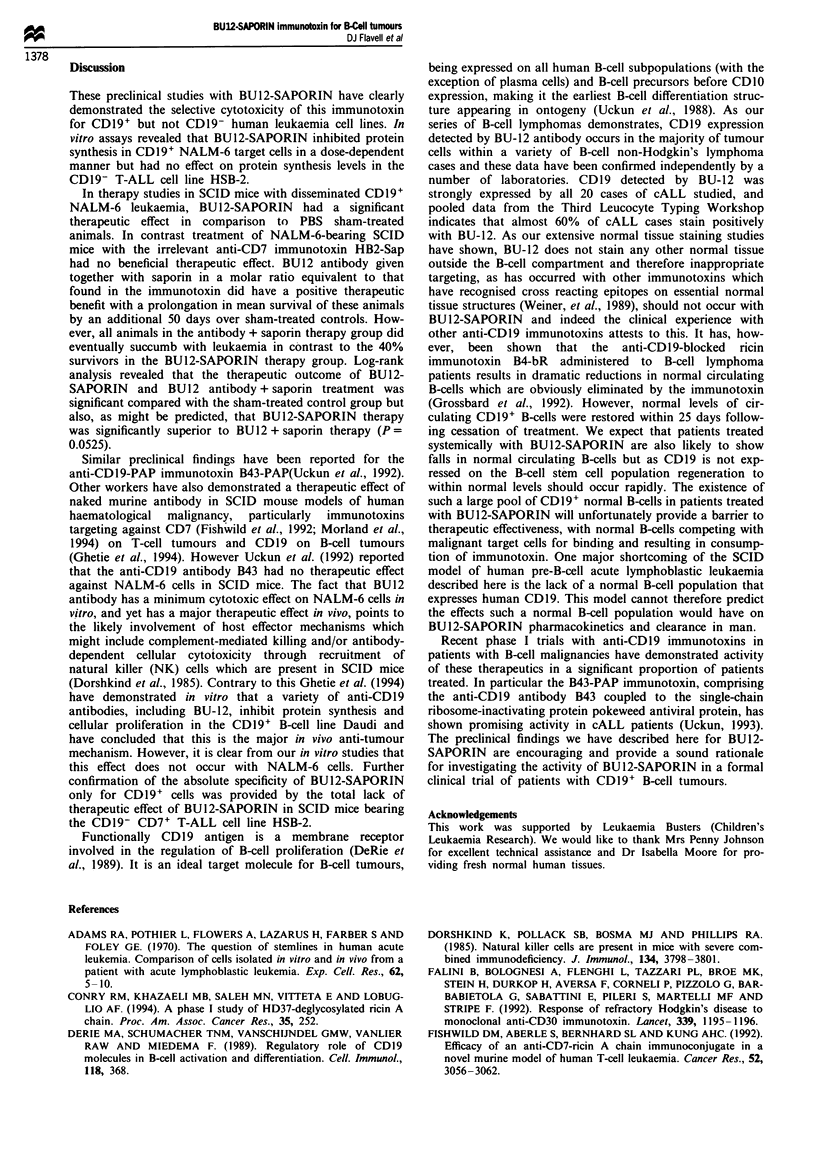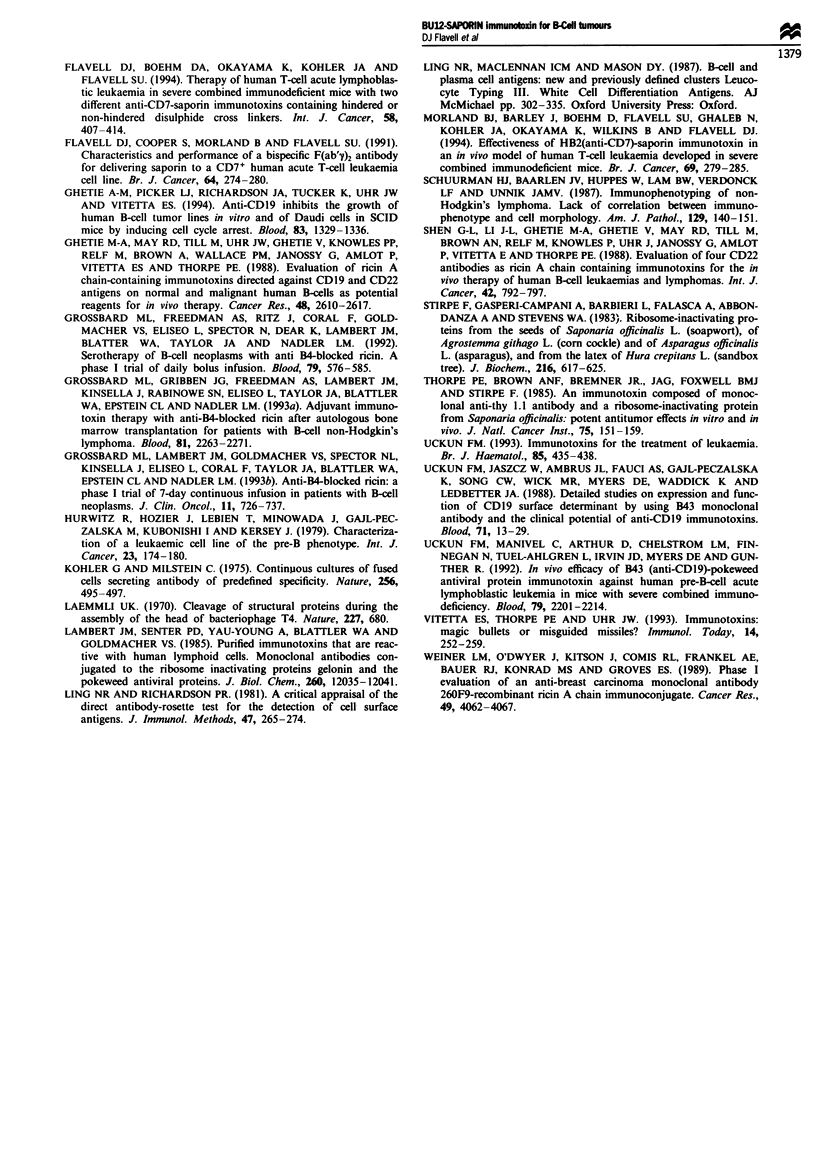# Preclinical studies with the anti-CD19-saporin immunotoxin BU12-SAPORIN for the treatment of human-B-cell tumours.

**DOI:** 10.1038/bjc.1995.517

**Published:** 1995-12

**Authors:** D. J. Flavell, S. U. Flavell, D. A. Boehm, L. Emery, A. Noss, N. R. Ling, P. R. Richardson, D. Hardie, D. H. Wright

**Affiliations:** Simon Flavell Leukaemia Research Laboratory, University Department of Pathology, Southampton General Hospital, UK.

## Abstract

**Images:**


British Journal of Cancer (1995) 72, 1373–1379

© 1995 Stockton Press All rights reserved 0007-0920/95 $12.00

### Preclinical studies with the anti-CD19-saporin immunotoxin BU12-SAPORIN for the treatment of human-B-cell tumours

DJ Flavell[1], SU Flavell[1], DA Boehm[1], L Emery[1], A Noss[1], NR Ling[2], PR Richardson[2], D Hardie[2] and DH Wright[1]

[1]*The Simon Flavell Leukaemia Research Laboratory, University Department of Pathology, Southampton General Hospital, Southampton S016 8EY, UK;* [2]*Department of Immunology, University of Birmingham Medical School, Vincent Drive, Birmingham UK.*

**Summary** The immunotoxin BU12-SAPORIN was constructed by covalently coupling the single-chain ribosome-inactivating protein saporin to the anti-CD19 monoclonal antibody BU12 via a disulphide linker using the heterobifunctional reagent SPDP. The immunoreactivity and specificity of BU12-SAPORIN was identical to that of unmodified native BU12 antibody. BU12-SAPORIN was selectively cytotoxic *in vitro* in a dose-dependent manner for the $CD19^+$ human common acute lymphoblastic leukaemia (cALL) cell line NALM-6 but exhibited no toxicity for the $CD19^-$ T-cell acute lymphoblastic leukaemia (T-ALL) cell line HSB-2. The survival of severe combined immunodeficient (SCID) mice with disseminated NALM-6 leukaemia was significantly prolonged compared with sham-treated control animals by a course of therapy with BU12-SAPORIN but not with the irrelevant anti-CD7 immunotoxin HB2-SAPORIN. BU12-SAPORIN had no therapeutic effect in SCID mice with disseminated $CD19^-$ HSB-2 leukaemia. These preclinical studies have clearly demonstrated the selective cytotoxicity of BU12-SAPORIN for $CD19^+$ target cells both *in vitro* and *in vivo*. This, taken together with the lack of expression of the CD19 molecule by any normal life-sustaining tissue and its ubiquitous and homogeneous expression by the majority of cALL and B-NHL cells, provides the rationale for undertaking a phase I trial of systemic therapy with BU12-SAPORIN.

**Keywords:** immunotoxin; anti-CD19; saporin; B-cell lymphoma; severe combined immunodeficient mouse

Various phase I/II clinical trials of systemic immunotoxin therapy for a variety of haematological malignancies have been completed or are currently in progress (Vitetta *et al.*, 1993). Two main target molecules, CD19 and CD22, have so far been exploited clinically for toxin delivery to malignant B cells. CD22 has proven an excellent target molecule in terms of the potency of immunotoxins made with antibodies recognising this B-cell structure (Shen *et al.*, 1988). However, expression of CD22 is quite heterogenous within B-cell tumours, often with only a small percentage of tumour cells expressing this molecule. This obviously limits the value of CD22 as target molecule in the context of immunotoxin-based therapies in which delivery of toxin to all individual tumour cells is required to ensure total ablation of the neoplastic clone.

CD19 on the other hand is a pan B-cell cell-surface differentiation structure that appears early in ontogeny and is expressed by the majority of pre-B and B-cell tumours with the exception of myeloma (Ling *et al.*, 1987). Moreover, in general CD19 is homogeneously expressed by the vast majority of cells within a given B-cell tumour which includes B-cell non-Hodgkin's lymphoma and pre-B-cell acute lymphoblastic leukaemia (Schuurman *et al.*, 1987). These characteristics make CD19 an ideal target molecule for toxin delivery to malignant B cells. However, immunotoxins constructed with anti-CD19 antibodies are generally less potent than those constructed with anti-CD22 antibodies (Ghetie *et al.*, 1988).

Three anti-CD19 immunotoxins have been reported in clinical use. B4-bR comprises the anti-CD19 antibody B4 coupled via a disulphide linker to blocked ricin (intact ricin in which the two galactose-binding domains of the B-chain have been chemically blocked) has been investigated in two separate phase I trials in patients with advanced B-cell tumours (Grossbard *et al.*, 1992, 1993*b*) and more recently in a phase I study in patients in complete remission with B-cell lymphoma following autologous bone marrow transplantation (Grossbard *et al.*, 1993*a*). HD37-dgR is an immunotoxin comprised of deglycosylated ricin A chain coupled via a hindered disulphide linker to the anti-CD19 antibody HD37 and has also been used in a phase I clinical study (Conry *et al.*, 1994). B43-PAP uses the single-chain ribosome-inactivating protein, pokeweed antiviral protein (PAP) coupled via a non-hindered disulphide bond to the B43 antibody. In formal phase I clinical trials, all three of these reagents have shown anti-tumour activity in a variable proportion of therapy-resistant patients, most notably B43-PAP in an ongoing phase I study has induced four complete responses in patients with multiply relapsed common acute lymphoblastic leukaemia (cALL). Saporin has never been targeted against CD19 in clinical trials in man though it has been used in immunotoxin form in four patients with Hodgkin's lymphoma using the anti-CD30 antibody Ber-H2 (Falini *et al.*, 1992), and major transient responses were observed.

We report here preclinical investigations with a new anti-CD19 immunotoxin BU12-SAPORIN which uses the single-chain ribosome-inactivating protein saporin coupled via a non-hindered disulphide bond to BU-12 antibody. BU12-SAPORIN is clearly demonstrated to be highly selective at delivering saporin only to $CD19^+$ B-cell lines *in vitro* and to be therapeutically effective *in vivo* in a SCID mouse model of human $CD19^+$ pre-B-cell acute lymphoblastic leukaemia. These preclinical studies provide the rationale for taking BU12-SAPORIN into a phase I clinical trial in patients with advanced therapy refractory B-cell tumours.

### Materials and methods

### *SCID mice*

Pathogen free BALB/c.C57BL/KaIgh-1/ICR scid/scid (SCID) mice of both sexes 6–10 weeks of age were produced from our own breeding colony and used in all the experimental work described here. The breeding colony is maintained under sterile conditions inside a laminar flow isolator and animals are housed on sterile bedding and provided with sterile water and food *ad libitum*. Animals for experimental

Correspondence: DJ Flavell
Received 21 April 1995; accepted 17 July 1995

use were transferred out from the isolator to autoclaved filter top microisolator cages and housed on sterile bedding as five single-sex animals per cage. These animals were also provided with sterile water and food *ad libitum* and all manipulations with these animals were carried out in a laminar flow hood by personnel using aseptic techniques.

*NALM-6 and HSB-2 human leukaemia cell lines*

The $CD19^+$ $CD7^-$ Pre-B leukaemia cell line NALM-6 was originally established from the peripheral blood of a 19-year-old male with non-T non-B acute lymphoblastic leukaemia (Hurwitz *et al*., 1979). The $CD7^+$ $CD19^-$ human cell line HSB-2 was originally established from peripheral blood leukaemic blasts from a 4-year-old paediatric patient with terminal T-cell acute lymphoblastic leukaemia (Adams *et al*., 1970). Both NALM-6 and HSB-2 cells were maintained in the logarithmic phase of growth in culture flasks containing antibiotic-free RPMI medium (Integra Biosciences, Northumbria, UK) containing 10% fetal calf serum and supplements of 2 mM sodium pyruvate and 2 mM glutamine (referred to hereafter as R10 medium) at 37°C under a humidified atmosphere of 5% carbon dioxide.

*Saporin production*

Seeds of the soapwort plant, *Saponaria officinalis*, were kindly supplied by Chiltern Seeds, Ulverston, Cumbria, UK. Saporin was extracted from seeds by the method of Stirpe *et al*. (1983) and purified to homogeneity by a combination of cation exchange chromatography on carboxymethyl-Sepharose and gel filtration on Sephacryl-S200HR. The final product gave a single band of 29 500 daltons on SDS–PAGE and was immunoreactive on ELISA with both polyclonal and monoclonal anti-saporin antisera.

*BU-12 antibody production*

The anti-CD19 antibody-producing hybridoma clone BU-12 was produced by the immunisation of two BALB/c mice with the Burkitt's lymphoma cell line EB4. Immune spleen cells from these animals were fused according to a modified method of Kohler and Milstein (1975) with the mouse X63 AG8 653 plasmacytoma cell line and resulting hybridomas selected in HAT medium. Screening of hybridoma culture supernatants on a range of T-, B-, myeloid and erythroid cell lines and on sections of normal human tonsil led to the selection of the pan-B-cell-reactive BU-12 hybridoma clone. The BU-12 antibody is of the $IgG_1$ subclass and was clustered and designated as an anti-CD19 antibody at the Third Leucocyte Typing Conference (B-cell section antibody no. 025) held in Oxford, UK in 1986 (Ling *et al*., 1987).

Bulk BU-12 antibody was manufactured by inoculating $5 \times 10^8$ BU-12 cells into an Endotronics Accusyst R hollow-fibre bioreactor (Endotronics, Minneapolis, MI, USA) according to the manufacturer's instructions with some minor modifications. Harvested antibody-containing culture supernatants were concentrated on a Sartorius cross-flow filtration apparatus (Sartorius, Epsom, UK) equipped with a 30 000 MW cut-off sanitisable cellulose acetate membrane. BU-12 antibody was purified to homogeneity from culture supernatants by a combination of ammonium sulphate precipitation, anion exchange chromatography on DEAE–Sepharose (Sigma, Poole, UK) and Sephacryl-S200HR (Sigma) gel filtration. Purified antibody gave a single band of 160 000 daltons on SDS–PAGE under non-reducing conditions and retained full immunoreactivity as demonstrated by flow cytometry.

*BU-12 SAPORIN immunotoxin construction*

BU-12 SAPORIN immunotoxin was constructed by conjugating the BU-12 antibody to saporin with the heterobifunctional cross linking reagent *N*-succinimidyl 3-(2-pyridyldithio) propionate (SPDP) as described by Thorpe *et al*. (1985). BU12-SAPORIN prepared in this way contained a non-hindered disulphide bond between antibody and saporin. Free antibody was removed from the immunoconjugates by carboxymethyl-Sepharose (Sigma) cation exchange chromatography as described previously (Lambert *et al*., 1985). Purified BU12-SAPORIN was dialysed into phosphate-buffered saline (PBS) pH 7.2, sterilised by passage through a 0.2 μm filter and stored deep frozen in aliquots at −80°C.

*HB2-Sap immunotoxin*

The preparation, characteristics and performance of the anti-CD7 immunotoxin HB2-Sap have been described in detail by us previously (Flavell *et al*., 1994; Morland *et al*., 1994).

*Sodium dodecyl sulphate polyacrylamide gel electophoresis (SDS–PAGE) analysis*

SDS–PAGE analysis according to the method of Laemmli (1970) was used to confirm purity of antibody, saporin and immunotoxins. Five per cent non-reducing SDS–PAGE gels with 3% stacking gels were routinely used for separations. Aliquots of 20 μg of each immunotoxin, HB2 antibody or saporin were added to individual wells following boiling in non-reducing sample buffer. Following electrophoresis gels were stained with Coomassie blue.

*Flow cytometry*

The binding of BU12-SAPORIN to NALM-6 or HSB-2 cells was confirmed and compared with that obtained for the native BU12 antibody by flow cytometry. One million NALM-6 or HSB-2 cells were incubated with concentrations of BU12-SAPORIN or native BU12 antibody diluted in PBS pH 7.2 (range 0.01–10 μg $ml^{-1}$) for 30 min at 4°C in the presence of 0.1% sodium azide. Negative control cells were incubated in PBS only. For staining samples of fresh cALL cells BU-12 antibody was conjugated directly to fluoroescein isothiocyanate (FITC) and binding measured directly by flow cytometry. For unconjugated BU-12 antibody, following staining, cells were washed twice in cold PBS containing 0.1% sodium azide and the cell pellets incubated for a further 30 min in 100 μl of FITC-labelled $Fab_2$ rabbit anti-mouse immunoglobulins (Sigma) diluted 1:20 in PBS. Cells were washed twice in PBS, resuspended in cold PBS and surface fluorescence analysed on a Becton Dickinson FACScan equipped with analytical software.

*Immunocytochemistry*

Fresh normal human tissues (listed in Table I) were snap frozen in liquid nitrogen and 5 μM thin sections cut. Sections were fixed in the dry acetone and stained with BU-12 antibody. Bound BU-12 was detected using a standard streptavidin immunoperoxidase system with diaminoazobenzidine as chromogen.

*Direct antibody rosette assay*

The direct antibody rosette test (Ling and Richardson, 1981) was used to determine CD19 expression (detected by BU-12 antibody) in a wide variety of fresh B-cell non-Hodgkin's lymphomas.

*Protein synthesis inhibition assay*

The ability of individual immunotoxins to inhibit protein synthesis in target cell lines was evaluated using a [$^3$H]leucine uptake assay described by us previously (Flavell *et al*., 1991). Briefly, triplicate cultures of $1 \times 10^5$ Target NALM-6 or HSB-2 cells were exposed to individual concentrations of BU-12 SAPORIN or HB2-Sap immunotoxin (range 0.0001–10 μg $ml^{-1}$), saporin (range 0.01–20 μg $ml^{-1}$) or BU-12 antibody (range 0.01–10 μg $ml^{-1}$) in R10 medium. Control

**Table I** Immunoreactivity of BU12 MAb and BU12-Sap IT on a range of normal human tissues

| *Tissue* | *Immunoreactivity* *BU12 MAb* | *BU12-Sap IT* |
|---|---|---|
| Adrenal | Negative | Negative |
| Bladder (including ureters) | Negative | Negative |
| Brain | Negative | Negative |
| Cartilage | Negative | Negative |
| Endothelium | Negative | Negative |
| Heart | Negative | Negative |
| Kidney | Negative | Negative |
| Large intestine | Negative | Negative |
| Liver | Negative | Negative |
| Lung | Negative | Negative |
| Lymph node | Positive B-cell follicles | Positive B-cell follicles |
| Pancreas | Negative | Negative |
| Peripheral nerve | Negative | Negative |
| Pituitary | Negative | Negative |
| Skin | Negative | Negative |
| Small intestine | Negative | Negative |
| Spinal cord | Negative | Negative |
| Spleen | Positive B-cell follicles | Positive B-cell follicles |
| Stomach | Negative | Negative |
| Testis | Negative | Negative |
| Thymus | Negative | Negative |
| Tonsil | Positive B-cell follicles | Positive B-cell follicles |
| Trachea | Negative | Negative |

cultures were incubated in R10 medium only. Cultures were incubated at 37°C for 48 h in a humidified atmosphere of 5% carbon dioxide/95% air. After this period of time 1 $\mu$Ci of [$^3$H]leucine was added to each culture and incubated for a further 14–16 h. Cells were harvested onto glass fibre discs using a Skatron Combi cell harvester (Skatron, Lier, Norway) and individual discs counted for radioactivity on a Packard 1600TR scintillation analyser (Canberra Packard, Pangbourne, UK). Results were expressed as a percentage of control values. The $IC_{50}$ value was calculated as the concentration of immunotoxin which inhibited protein synthesis in target cells by 50% relative to controls.

*Establishment of NALM-6 or HSB-2 leukaemia in SCID mice*

NALM-6 or HSB-2 human leukaemia cells were injected intravenously into SCID mice in a 200 $\mu$l volume of R10 medium.

*Therapy of leukaemia-bearing SCID mice with immunotoxins*

Groups of ten (five male and five female) SCID mice, 6–10 weeks of age received $2 \times 10^6$ NALM-6 or HSB-2 cells intravenously. Seven days after injection of tumour cells appropriate groups received $3 \times 10\ \mu g$ doses of BU12-SAPORIN, HB2-Sap, BU-12 antibody + saporin ($8\ \mu g + 2\ \mu g$) or sham therapy with PBS, each injection being given intravenously on alternate days (i.e. 7, 9 and 11 days after injection of tumour cells). Each injection was given in 200 $\mu$l of PBS as solvent. Animals were observed on a daily basis and those showing signs of hind leg paralysis (SCID-NALM-6 animals only) or which became moribund were painlessly killed and subjected to post-mortem examination. All major organs were removed for histopathological examination to confirm the presence of disease.

### Results

*SDS–PAGE analysis of BU12, saporin and BU12-SAPORIN*

Results of SDS–PAGE analysis of BU12-SAPORIN immunotoxin under non-reducing conditions are shown in Figure 1. Two bands of 190 and 220 kDa were detected corresponding to species of immunotoxin comprising one antibody molecule coupled to one or two saporin molecules respec-

**Figure 1** SDS–PAGE analysis on a non-reducing 5% separating gel of BU12-SAPORIN (lane 1), BU12 antibody (lane 2) and saporin (lane 3). Molecular weights shown are in kDa.

tively (lane 1). The relative proportions of the 190 and 220 kDa immunotoxin species was approximately 3:1. Less than 1% free BU12 antibody or saporin was detectable in the BU12-SAPORIN. BU12 antibody (lane 2) gave a single band of 160 kDa and saporin (lane 3) a single band of 29.5 kDa.

*Binding characteristics of BU12 and BU12-SAPORIN to NALM-6 and HSB-2 cells.*

Results of flow cytometric analysis of BU12 antibody and BU12-SAPORIN IT binding to NALM-6 cells are shown in Figure 2. BU12 and BU12-SAPORIN stained NALM-6 cells with equal intensity when used at a saturating concentration of 10 $\mu g\ ml^{-1}$ or a subsaturating concentration of 1 $\mu g\ ml^{-1}$. BU12 or BU12-SAPORIN immunotoxin did not stain the $CD19^-$ cell line HSB-2 (data not shown).

*Normal tissue staining with BU12 and BU12-SAPORIN*

The staining characteristics of BU12 antibody and BU12-SAPORIN immunotoxin were studied on a wide variety of normal human tissues and the results are listed in Table I. Both performed identically and stained only B lymphocytes within the B-cell compartments of primary and secondary lymphoid tissues. No other normal tissue was seen to stain with either.

*Reactivity of BU-12 with malignant B cells*

Tumour cells from all 400 cases of B-cell chronic lymphocytic leukaemia, 20 cases of pre-B-cell acute lymphoblastic leukaemia and 159 cases of B-cell non-Hodgkin's lymphoma were positive for CD19 expression detected with BU12 antibody.

*Protein synthesis inhibition in target and non-target cells by BU12-SAPORIN*

BU12-SAPORIN inhibited protein synthesis in target NALM-6 cells in a dose-dependent manner (Figure 3). The $IC_{50}$ value was achieved at a BU12-SAPORIN concentration of 0.0069 $\mu g\ ml^{-1}$. Native BU12 antibody used over the same concentration range had virtually no effect on protein synthesis levels in NALM-6 cells. Similarly, the irrelevant anti-CD7 immunotoxin HB2-Sap used over the same concentration range as BU12-SAPORIN had no significant effect on protein synthesis levels in NALM-6 cells. The $IC_{50}$ value for saporin alone was 2.4 $\mu g\ ml^{-1}$ and therefore the calculated increase in cytotoxicity of BU12-SAPORIN over native saporin was 347-fold.

Figure 4 shows protein synthesis levels in the $CD19^-$ $CD7^+$ T-ALL cell line HSB-2 exposed to increasing concentrations (0.001–10 $\mu g\ ml^{-1}$) of BU12-SAPORIN or HB2-Sap. BU12-SAPORIN did not inhibit protein synthesis in HSB-2 cells at any of the concentrations investigated. As expected, the anti-CD7 immunotoxin HB2-Sap, inhibited protein synthesis in $CD7^+$ HSB-2 cells in a dose-dependent manner with an achieved $IC_{50}$ value of 0.014 $\mu g\ ml^{-1}$.

**Figure 3** Inhibition of protein synthesis in $CD19^+$ $CD7^-$ NALM-6 target cells by BU12-SAPORIN (●——●), HB2-SAP (O——O), BU12 antibody (▲——▲) or saporin (▼——▼). Error bars represent one standard deviation from the mean result obtained for triplicate cultures for each data point.

**Figure 2** Flow cytometry profiles of $CD19^+$ NALM-6 cells stained with BU12 antibody at 10 $\mu g\ ml^{-1}$ (**a**) or 1 $\mu g\ ml^{-1}$ (**b**) or with BU12-SAPORIN immunotoxin at 10 $\mu g\ ml^{-1}$ (**c**) or 1 $\mu g\ ml^{-1}$ (**d**). Unstained control NALM-6 cells are shown as a broken line. Figures in paraentheses refer to the mean fluorescent intensity in arbitrary units.

Figure 4 Inhibition of protein synthesis in $CD19^-$ $CD7^+$ HSB-2 target cells by BU12-SAPORIN (●——●), HB2-SAP (O——O) or saporin (▼——▼). Error bars represent one standard deviation from the mean result obtained for triplicate cultures for each data point.

Figure 5 Survival of groups of SCID mice injected intravenously with various numbers of NALM-6 leukaemia cells. Number of NALM-6 cells ————, $10^7$; — — —, $10^6$; - - - -, $10^5$; - - - -, $10^4$.

### *NALM-6 and HSB-2 leukaemia in SCID mice*

SCID mice injected intravenously with NALM-6 cells developed hind leg paralysis which progressed to quadraplegia, leading to eventual death. In previous studies we demonstrated that all animals that developed hind leg paralysis became moribund and died with NALM-6 cells infiltrating the CNS and bone marrow 7–10 days following onset of paralysis. Near-identical findings for the behaviour of NALM-6 tumour cells in SCID mice have been reported by Uckun *et al.* (1992). For humane purposes we painlessly killed experimental animals in these studies when they first showed signs of paralysis and the survival figures expressed thus represent survival up to this point. Figure 5 shows the direct relationship between the number of NALM-6 cells injected and survival time, with animals receiving the greatest number of cells surviving for the shortest period.

We have previously described in detail the characteristics of acute T-cell HSB-2 leukaemia in SCID mice (Morland *et al.*, 1994). Animals with disseminated HSB-2 leukaemia have multiorgan involvement but generally do not develop neurological problems because HSB-2 tumour cells do not infiltrate the CNS as extensively as NALM-6 cells. Animals with HSB-2 leukaemia thus tend to develop a wasting-type disease and become moribund as essential organs are infiltrated.

### *Therapy of SCID-NALM-6 mice with BU12-SAPORIN*

Figure 6a shows the survival of groups of SCID mice injected i.v. with $2 \times 10^6$ NALM-6 cells followed by sham therapy with PBS, BU12-SAPORIN, the irrelevant anti-CD7 saporin immunotoxin HB2-Sap or native BU12 antibody + saporin. Sham-treated control animals had a median survival time of 40.2 days with all animals dead within this group by 45 days. Treatment with BU12-SAPORIN led to a significant prolongation in survival (sham-treated controls *vs* BU12-SAPORIN therapy $P=$ 0.000723 by log-rank analysis) of SCID-NALM-6 mice with 40% of animals alive and apparently disease free at the termination of the experiment at 110 days. Treatment with unconjugated BU12 antibody + saporin also had a significant therapeutic effect (sham-treated controls *vs* BU12 + saporin treatment $P = 0.00413$), animals within this treatment group having a median survival of 63.3 days but with all animals dead by 95 days. Comparison of the BU12-SAPORIN IT treatment group with the BU12 + saporin group by log-rank analysis revealed a significant difference between the two ($P = 0.0525$) with BU12-SAPORIN treatment being obviously superior. The irrelevant anti-CD7 immunotoxin HB2-SAP had no therapeutic effect in SCID-NALM-6 mice, animals within this group dying at approximately the same rate as sham-treated controls with a median survival time of 41.3 days.

Figure 6 Survival curves for groups of SCID mice following i.v. challenge with $2 \times 10^6$ NALM-6 (a) or HSB-2 cells (b) followed 7 days later by treatment on 3 alternate days with BU12-SAPORIN (– – –), BU12 antibody + saporin (......), HB2-Sap (- - - -) or PBS (————).

Therapy studies with BU12-SAPORIN were also undertaken in SCID mice bearing the $CD19^-$ $CD7^+$ T-ALL cell line HSB-2 and the results are shown in Figure 6b. BU12-SAPORIN had no therapeutic effect in this SCID-HSB-2 model, with treated animals dying at approximately the same rate as sham-treated controls (median survival times of 57.6 days and 58 days respectively). In contrast, as reported by us previously, treatment with the anti-CD7 immunotoxin HB2-Sap led to a significantly prolonged survival with 90% of animals within this therapy group alive at 150 days (Flavell *et al.*, 1994).

### Discussion

These preclinical studies with BU12-SAPORIN have clearly demonstrated the selective cytotoxicity of this immunotoxin for CD19$^+$ but not CD19$^-$ human leukaemia cell lines. *In vitro* assays revealed that BU12-SAPORIN inhibited protein synthesis in CD19$^+$ NALM-6 target cells in a dose-dependent manner but had no effect on protein synthesis levels in the CD19$^-$ T-ALL cell line HSB-2.

In therapy studies in SCID mice with disseminated CD19$^+$ NALM-6 leukaemia, BU12-SAPORIN had a significant therapeutic effect in comparison to PBS sham-treated animals. In contrast treatment of NALM-6-bearing SCID mice with the irrelevant anti-CD7 immunotoxin HB2-Sap had no beneficial therapeutic effect. BU12 antibody given together with saporin in a molar ratio equivalent to that found in the immunotoxin did have a positive therapeutic benefit with a prolongation in mean survival of these animals by an additional 50 days over sham-treated controls. However, all animals in the antibody + saporin therapy group did eventually succumb with leukaemia in contrast to the 40% survivors in the BU12-SAPORIN therapy group. Log-rank analysis revealed that the therapeutic outcome of BU12-SAPORIN and BU12 antibody + saporin treatment was significant compared with the sham-treated control group but also, as might be predicted, that BU12-SAPORIN therapy was significantly superior to BU12 + saporin therapy ($P = 0.0525$).

Similar preclinical findings have been reported for the anti-CD19-PAP immunotoxin B43-PAP(Uckun *et al.*, 1992). Other workers have also demonstrated a therapeutic effect of naked murine antibody in SCID mouse models of human haematological malignancy, particularly immunotoxins targeting against CD7 (Fishwild *et al.*, 1992; Morland *et al.*, 1994) on T-cell tumours and CD19 on B-cell tumours (Ghetie *et al.*, 1994). However Uckun *et al.* (1992) reported that the anti-CD19 antibody B43 had no therapeutic effect against NALM-6 cells in SCID mice. The fact that BU12 antibody has a minimum cytotoxic effect on NALM-6 cells *in vitro*, and yet has a major therapeutic effect *in vivo*, points to the likely involvement of host effector mechanisms which might include complement-mediated killing and/or antibody-dependent cellular cytotoxicity through recruitment of natural killer (NK) cells which are present in SCID mice (Dorshkind *et al.*, 1985). Contrary to this Ghetie *et al.* (1994) have demonstrated *in vitro* that a variety of anti-CD19 antibodies, including BU-12, inhibit protein synthesis and cellular proliferation in the CD19$^+$ B-cell line Daudi and have concluded that this is the major *in vivo* anti-tumour mechanism. However, it is clear from our *in vitro* studies that this effect does not occur with NALM-6 cells. Further confirmation of the absolute specificity of BU12-SAPORIN only for CD19$^+$ cells was provided by the total lack of therapeutic effect of BU12-SAPORIN in SCID mice bearing the CD19$^-$ CD7$^+$ T-ALL cell line HSB-2.

Functionally CD19 antigen is a membrane receptor involved in the regulation of B-cell proliferation (DeRie *et al.*, 1989). It is an ideal target molecule for B-cell tumours, being expressed on all human B-cell subpopulations (with the exception of plasma cells) and B-cell precursors before CD10 expression, making it the earliest B-cell differentiation structure appearing in ontogeny (Uckun *et al.*, 1988). As our series of B-cell lymphomas demonstrates, CD19 expression detected by BU-12 antibody occurs in the majority of tumour cells within a variety of B-cell non-Hodgkin's lymphoma cases and these data have been confirmed independently by a number of laboratories. CD19 detected by BU-12 was strongly expressed by all 20 cases of cALL studied, and pooled data from the Third Leucocyte Typing Workshop indicates that almost 60% of cALL cases stain positively with BU-12. As our extensive normal tissue staining studies have shown, BU-12 does not stain any other normal tissue outside the B-cell compartment and therefore inappropriate targeting, as has occurred with other immunotoxins which have recognised cross reacting epitopes on essential normal tissue structures (Weiner, *et al.*, 1989), should not occur with BU12-SAPORIN and indeed the clinical experience with other anti-CD19 immunotoxins attests to this. It has, however, been shown that the anti-CD19-blocked ricin immunotoxin B4-bR administered to B-cell lymphoma patients results in dramatic reductions in normal circulating B-cells which are obviously eliminated by the immunotoxin (Grossbard *et al.*, 1992). However, normal levels of circulating CD19$^+$ B-cells were restored within 25 days following cessation of treatment. We expect that patients treated systemically with BU12-SAPORIN are also likely to show falls in normal circulating B-cells but as CD19 is not expressed on the B-cell stem cell population regeneration to within normal levels should occur rapidly. The existence of such a large pool of CD19$^+$ normal B-cells in patients treated with BU12-SAPORIN will unfortunately provide a barrier to therapeutic effectiveness, with normal B-cells competing with malignant target cells for binding and resulting in consumption of immunotoxin. One major shortcoming of the SCID model of human pre-B-cell acute lymphoblastic leukaemia described here is the lack of a normal B-cell population that expresses human CD19. This model cannot therefore predict the effects such a normal B-cell population would have on BU12-SAPORIN pharmacokinetics and clearance in man.

Recent phase I trials with anti-CD19 immunotoxins in patients with B-cell malignancies have demonstrated activity of these therapeutics in a significant proportion of patients treated. In particular the B43-PAP immunotoxin, comprising the anti-CD19 antibody B43 coupled to the single-chain ribosome-inactivating protein pokeweed antiviral protein, has shown promising activity in cALL patients (Uckun, 1993). The preclinical findings we have described here for BU12-SAPORIN are encouraging and provide a sound rationale for investigating the activity of BU12-SAPORIN in a formal clinical trial of patients with CD19$^+$ B-cell tumours.

**Acknowledgements**
This work was supported by Leukaemia Busters (Children's Leukaemia Research). We would like to thank Mrs Penny Johnson for excellent technical assistance and Dr Isabella Moore for providing fresh normal human tissues.

### References

ADAMS RA, POTHIER L, FLOWERS A, LAZARUS H, FARBER S AND FOLEY GE. (1970). The question of stemlines in human acute leukemia. Comparison of cells isolated *in vitro* and *in vivo* from a patient with acute lymphoblastic leukemia. *Exp. Cell. Res.*, **62**, 5-10.

CONRY RM, KHAZAELI MB, SALEH MN, VITTETA E AND LOBUGLIO AF. (1994). A phase I study of HD37-deglycosylated ricin A chain. *Proc. Am. Assoc. Cancer Res.*, **35**, 252.

DERIE MA, SCHUMACHER TNM, VANSCHIJNDEL GMW, VANLIER RAW AND MIEDEMA F. (1989). Regulatory role of CD19 molecules in B-cell activation and differentiation. *Cell. Immunol.*, **118**, 368.

DORSHKIND K, POLLACK SB, BOSMA MJ AND PHILLIPS RA. (1985). Natural killer cells are present in mice with severe combined immunodeficiency. *J. Immunol.*, **134**, 3798-3801.

FALINI B, BOLOGNESI A, FLENGHI L, TAZZARI PL, BROE MK, STEIN H, DURKOP H, AVERSA F, CORNELI P, PIZZOLO G, BARBABIETOLA G, SABATTINI E, PILERI S, MARTELLI MF AND STRIPE F. (1992). Response of refractory Hodgkin's disease to monoclonal anti-CD30 immunotoxin. *Lancet*, **339**, 1195-1196.

FISHWILD DM, ABERLE S, BERNHARD SL AND KUNG AHC. (1992). Efficacy of an anti-CD7-ricin A chain immunoconjugate in a novel murine model of human T-cell leukaemia. *Cancer Res.*, **52**, 3056-3062.

FLAVELL DJ, BOEHM DA, OKAYAMA K, KOHLER JA AND FLAVELL SU. (1994). Therapy of human T-cell acute lymphoblastic leukaemia in severe combined immunodeficient mice with two different anti-CD7-saporin immunotoxins containing hindered or non-hindered disulphide cross linkers. *Int. J. Cancer*, **58**, 407-414.

FLAVELL DJ, COOPER S, MORLAND B AND FLAVELL SU. (1991). Characteristics and performance of a bispecific $F(ab'\gamma)_2$ antibody for delivering saporin to a $CD7^+$ human acute T-cell leukaemia cell line. *Br. J. Cancer*, **64**, 274-280.

GHETIE A-M, PICKER LJ, RICHARDSON JA, TUCKER K, UHR JW AND VITETTA ES. (1994). Anti-CD19 inhibits the growth of human B-cell tumor lines *in vitro* and of Daudi cells in SCID mice by inducing cell cycle arrest. *Blood*, **83**, 1329-1336.

GHETIE M-A, MAY RD, TILL M, UHR JW, GHETIE V, KNOWLES PP, RELF M, BROWN A, WALLACE PM, JANOSSY G, AMLOT P, VITETTA ES AND THORPE PE. (1988). Evaluation of ricin A chain-containing immunotoxins directed against CD19 and CD22 antigens on normal and malignant human B-cells as potential reagents for *in vivo* therapy. *Cancer Res.*, **48**, 2610-2617.

GROSSBARD ML, FREEDMAN AS, RITZ J, CORAL F, GOLDMACHER VS, ELISEO L, SPECTOR N, DEAR K, LAMBERT JM, BLATTER WA, TAYLOR JA AND NADLER LM. (1992). Serotherapy of B-cell neoplasms with anti B4-blocked ricin. A phase I trial of daily bolus infusion. *Blood*, **79**, 576-585.

GROSSBARD ML, GRIBBEN JG, FREEDMAN AS, LAMBERT JM, KINSELLA J, RABINOWE SN, ELISEO L, TAYLOR JA, BLATTLER WA, EPSTEIN CL AND NADLER LM. (1993*a*). Adjuvant immunotoxin therapy with anti-B4-blocked ricin after autologous bone marrow transplantation for patients with B-cell non-Hodgkin's lymphoma. *Blood*, **81**, 2263-2271.

GROSSBARD ML, LAMBERT JM, GOLDMACHER VS, SPECTOR NL, KINSELLA J, ELISEO L, CORAL F, TAYLOR JA, BLATTLER WA, EPSTEIN CL AND NADLER LM. (1993*b*). Anti-B4-blocked ricin: a phase I trial of 7-day continuous infusion in patients with B-cell neoplasms. *J. Clin. Oncol.*, **11**, 726-737.

HURWITZ R, HOZIER J, LEBIEN T, MINOWADA J, GAJL-PECZALSKA M, KUBONISHI I AND KERSEY J. (1979). Characterization of a leukaemic cell line of the pre-B phenotype. *Int. J. Cancer*, **23**, 174-180.

KOHLER G AND MILSTEIN C. (1975). Continuous cultures of fused cells secreting antibody of predefined specificity. *Nature*, **256**, 495-497.

LAEMMLI UK. (1970). Cleavage of structural proteins during the assembly of the head of bacteriophage T4. *Nature*, **227**, 680.

LAMBERT JM, SENTER PD, YAU-YOUNG A, BLATTLER WA AND GOLDMACHER VS. (1985). Purified immunotoxins that are reactive with human lymphoid cells. Monoclonal antibodies conjugated to the ribosome inactivating proteins gelonin and the pokeweed antiviral proteins. *J. Biol. Chem.*, **260**, 12035-12041.

LING NR AND RICHARDSON PR. (1981). A critical appraisal of the direct antibody-rosette test for the detection of cell surface antigens. *J. Immunol. Methods*, **47**, 265-274.

LING NR, MACLENNAN ICM AND MASON DY. (1987). B-cell and plasma cell antigens: new and previously defined clusters Leucocyte Typing III. White Cell Differentiation Antigens. AJ McMichael pp. 302-335. Oxford University Press: Oxford.

MORLAND BJ, BARLEY J, BOEHM D, FLAVELL SU, GHALEB N, KOHLER JA, OKAYAMA K, WILKINS B AND FLAVELL DJ. (1994). Effectiveness of HB2(anti-CD7)-saporin immunotoxin in an *in vivo* model of human T-cell leukaemia developed in severe combined immunodeficient mice. *Br. J. Cancer*, **69**, 279-285.

SCHUURMAN HJ, BAARLEN JV, HUPPES W, LAM BW, VERDONCK LF AND UNNIK JAMV. (1987). Immunophenotyping of non-Hodgkin's lymphoma. Lack of correlation between immunophenotype and cell morphology. *Am. J. Pathol.*, **129**, 140-151.

SHEN G-L, LI J-L, GHETIE M-A, GHETIE V, MAY RD, TILL M, BROWN AN, RELF M, KNOWLES P, UHR J, JANOSSY G, AMLOT P, VITETTA E AND THORPE PE. (1988). Evaluation of four CD22 antibodies as ricin A chain containing immunotoxins for the *in vivo* therapy of human B-cell leukaemias and lymphomas. *Int. J. Cancer*, **42**, 792-797.

STIRPE F, GASPERI-CAMPANI A, BARBIERI L, FALASCA A, ABBONDANZA A AND STEVENS WA. (1983). Ribosome-inactivating proteins from the seeds of *Saponaria officinalis* L. (soapwort), of *Agrostemma githago* L. (corn cockle) and of *Asparagus officinalis* L. (asparagus), and from the latex of *Hura crepitans* L. (sandbox tree). *J. Biochem.*, **216**, 617-625.

THORPE PE, BROWN ANF, BREMNER JR., JAG, FOXWELL BMJ AND STIRPE F. (1985). An immunotoxin composed of monoclonal anti-thy 1.1 antibody and a ribosome-inactivating protein from *Saponaria officinalis:* potent antitumor effects *in vitro* and *in vivo*. *J. Natl. Cancer Inst.*, **75**, 151-159.

UCKUN FM. (1993). Immunotoxins for the treatment of leukaemia. *Br. J. Haematol.*, **85**, 435-438.

UCKUN FM, JASZCZ W, AMBRUS JL, FAUCI AS, GAJL-PECZALSKA K, SONG CW, WICK MR, MYERS DE, WADDICK K AND LEDBETTER JA. (1988). Detailed studies on expression and function of CD19 surface determinant by using B43 monoclonal antibody and the clinical potential of anti-CD19 immunotoxins. *Blood*, **71**, 13-29.

UCKUN FM, MANIVEL C, ARTHUR D, CHELSTROM LM, FINNEGAN N, TUEL-AHLGREN L, IRVIN JD, MYERS DE AND GUNTHER R. (1992). *In vivo* efficacy of B43 (anti-CD19)-pokeweed antiviral protein immunotoxin against human pre-B-cell acute lymphoblastic leukemia in mice with severe combined immunodeficiency. *Blood*, **79**, 2201-2214.

VITETTA ES, THORPE PE AND UHR JW. (1993). Immunotoxins: magic bullets or misguided missiles? *Immunol. Today*, **14**, 252-259.

WEINER LM, O'DWYER J, KITSON J, COMIS RL, FRANKEL AE, BAUER RJ, KONRAD MS AND GROVES ES. (1989). Phase I evaluation of an anti-breast carcinoma monoclonal antibody 260F9-recombinant ricin A chain immunoconjugate. *Cancer Res.*, **49**, 4062-4067.